# Anévrisme de l'artère splénique rompu dans l'estomac: traitement chirurgical après échec d'une tentative d'embolisation

**DOI:** 10.11604/pamj.2015.20.66.3667

**Published:** 2015-01-26

**Authors:** Imad Lachhab, Amine Benkabbou, Amine Souadka, Haj Omar El Malki, Raouf Mohsine, Lahcen Ifrine, Abdelkader Belkouchi

**Affiliations:** 1 Service de Chirurgie Générale A, Hôpital Ibn Sina–CHU Rabat, Faculté de Médecine et de Pharmacie Mohamed V, Rabat, Maroc

**Keywords:** Anévrisme, artère splénique, hémorragie digestive, aneurysm, Splenic artery, gastrointestinal bleeding

## Abstract

L'anévrisme de l'artère splénique (AAS) est une entité pathologique rare le plus souvent asymptomatique. Nous rapportons le cas d'un patient âgé de 60 ans, hypertendu qui s'est présenté aux urgences pour un épisode d'hématémèse sans retentissement hémodynamique. Un bilan complet comportant un Angioscanner abdominal a mis en évidence un anévrisme de l'artère splénique refoulant la paroi postérieure de l'estomac en avant. Le diagnostic d'anévrisme de l'artère splénique rompu dans l'estomac a été posé et un traitement endovasculaire à type d'embolisation par coils effectué. Au 5^ème^ jour post embolisation, le patient nous a été référé pour une persistance de mélénas. Un traitement chirurgical a été décidé. La mise à plat de l'anévrisme a permis d’évacuer les coils et le thrombus. L'objectif de cette observation est de montrer que l'embolisation d'un AAS rompu dans l'estomac a été une cause de retard thérapeutique qui pourrait être fatal pour le patient. Le traitement de référence est la cure chirurgicale de l'AAS par voie conventionnelle sans rétablissement de la continuité artérielle splénique, sans splénectomie et avec suture de l'orifice digestif.

## Introduction

L'anévrisme de l'artère splénique (AAS) occupe le premier rang des anévrismes des artères viscérales et le troisième rang des anévrismes intra abdominaux après l'anévrisme de l'aorte et des artères iliaques [1, 2]. C'est une entité rare avec une incidence comprise entre 0,01-0,2% [3]. L'AAS est le plus souvent asymptomatique mais il peut être révélé par une hémorragie digestive qui traduit une rupture dans l'estomac. Nous rapportons une observation d'AAS rompu dans l'estomac traité chirurgicalement après échec d'une tentative de traitement endovasculaire par embolisation.

## Patient et observation

**Figure 1:**
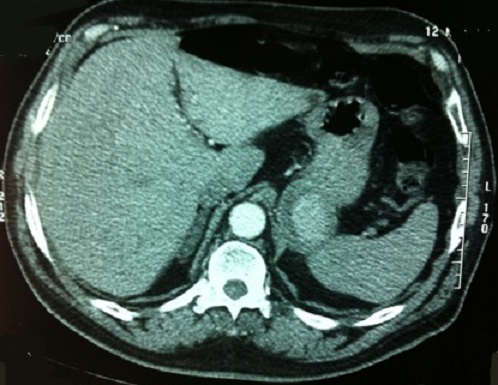
TDM abdominale montrant un anévrisme de l'artère splénique

**Figure 2:**
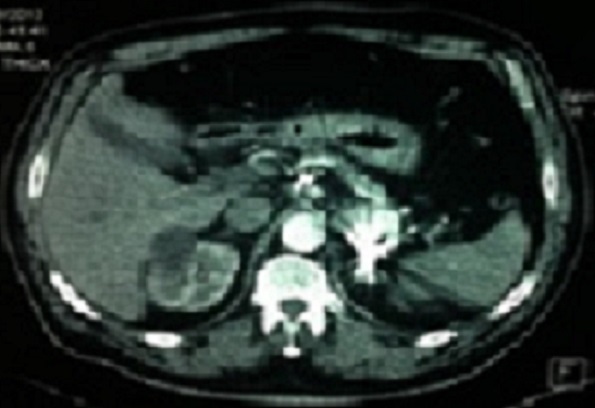
TDM abdominale montrant une embolisation de l'anévrisme de l'artère splénique par coils

**Figure 3:**
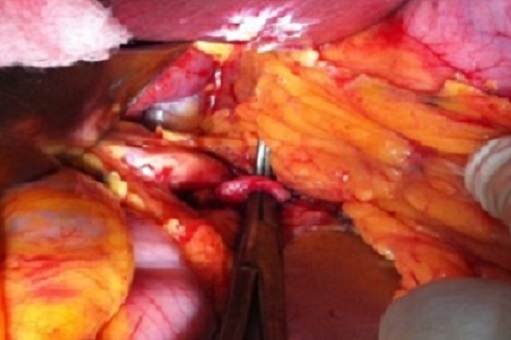
Ligature de l'artère splénique en aval de l'anévrisme

**Figure 4:**
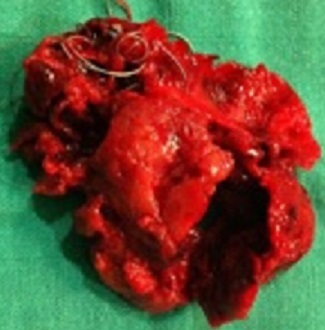
Évacuation du contenu de l'anévrisme thrombus et coils

**Figure 5:**
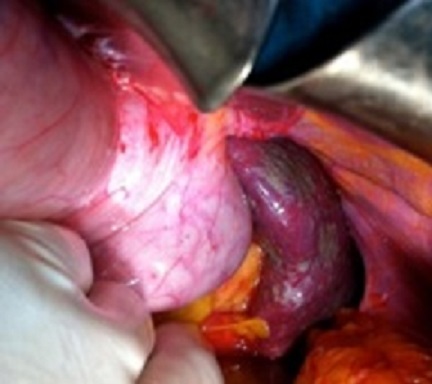
Absence d'infarcissement splénique en fin d'intervention

Un homme de 60 ans avec des facteurs de risques vasculaires à type d'obésité (BMI = 29,7), d'hypertension artérielle et de tabagisme chronique (30 paquets-an) s'est présenté aux urgences pour un épisode d'hématémèse sans retentissement hémodynamique. Une endoscopie oeso-gastroduodénale avec biopsies a montré des lésions de gastrite aspécifique traitée par inhibiteurs de la pompe à proton (IPP) sans argument pour une maladie ulcéreuse ou une hypertension portale. Quatre jours plus tard le patient a présenté un deuxième épisode d'hématémèse avec hypotension et déglobulisation justifiant une hospitalisation en urgence avec transfusion sanguine de 4 culots globulaires. Après restauration de l’état hémodynamique, un bilan comportant un angioscanner abdominal a mis en évidence un anévrisme de l'artère splénique (AAS) mesurant 46 × 56 mm refoulant le corps du pancréas en arrière et la paroi postérieure de l'estomac en avant (Figure 1). Le diagnostic d'anévrisme de l'artère splénique rompu dans l'estomac a été posé et un traitement endovasculaire à type d'embolisation de l'anévrisme par coils effectué (Figure 2). Au 5^ème^ jour post-embolisation, le patient nous a été référé pour une persistance de méléna sans instabilité hémodynamique mais avec une déglobulisation progressive justifiant une seconde transfusion de 2 culots globulaires. Un traitement chirurgical en urgence par voie conventionnelle (laparotomie sous costale gauche) a été décidé. L'exploration a retrouvé des anses intestinales pleines de sang avec un foie d'aspect morphologique normal. L'accès à l'arrière cavité des épiploons après décollement colo-épiploique a permis de contrôler puis de lier l'artère splénique de part et d'autre de l'AAS situé entre le bord supérieur du corps de pancréas et la partie postérieur du corps gastrique (Figure 3). Les artères hépatiques, coronaires stomachiques, et le tronc cœliaque n’étaient pas concernés par l'anévrisme. La mise à plat de l'AAS a permis d’évacuer les coils et le thrombus (Figure 4) et d'oblitérer électivement au mono filament non résorbable fin (5-0) les collatérales artérielles pancréatiques alimentant l'anévrisme. Le diamètre de l'orifice de rupture de l'AAS dans l'estomac était de 10mm avec des bords remaniés et un trajet en chicane dans la paroi gastrique. Cet orifice a été avivé et suturé par des points séparés de fil résorbable (3-0). Aucun infarcissement de la rate n’était noté en fin d'intervention (Figure 5). Un drain a été mis au contact de la suture gastrique. Les suites ont été marquées par une fistule pancréatique asymptomatique (lipase dans le liquide de drainage= 30 000 UI/L) extériorisée par le drain. Le patient a quitté le service à J10 postopératoire sous IPP 40mg/J. La fistule pancréatique s'est tarie progressivement et le drain a pu être retiré à J42 postopératoire. L’étude histologique de la paroi anévrismale a montré la présence de micro abcès. Le patient est actuellement asymptomatique après 1 an d’évolution.

## Discussion

Les anévrismes de l'artère splénique (AAS) sont le plus souvent asymptomatiques et leur découverte se fait fortuitement lors de la réalisation d'un examen morphologique abdominal indiqué pour une autre pathologie [4]. L'AAS est quatre fois plus fréquent chez la femme avec une moyenne d’âge de 50 à 60 ans [5]. L’étiologie précise des AAS demeure indéterminée mais ils sont associés à des situations telles que la grossesse, la cirrhose et/ou l'hypertension portale [4]. Dans 80%, des cas, on retrouve au scanner des signes d'athérosclérose [5]. Notre patient avait de nombreux facteurs de risque vasculaires: obésité, hypertension artérielle et tabagisme. Lorsque l'AAS est symptomatique, la douleur abdominale est le signe le plus fréquent [6]. L'hémorragie digestive traduisant une rupture de l'AAS dans le tube digestif est rare et peut être inaugurale [3, 6]. L’échographie couplée au doppler peut faire le diagnostic d'AAS [7] et doit être complétée par un angioscanner abdominal. Les séries sans injection permettent d’évaluer les calcifications des parois artérielles tandis que les séries au temps artériel après injection permettent une reconstruction précise de l'anévrisme et de l'anatomie artérielle. En cas de contre indication au scanner, l'angio IRM est une option valide pour identifier et localiser un AAS [8]. Au plan diagnostic, ces examens morphologiques non invasifs tendent à remplacer l'artériographie qui se limite à l'analyse endoluminale [7]. Le risque évolutif principal d'un anévrisme est la rupture. Ce risque atteint 28% lorsque la taille de l'anévrisme est supérieure à 50mm [5]. Les autres complications potentielles des AAS sont l'infarcissement splénique ou l'ictère par compression biliaire [5]. Les indications de traitement concernent tous les AAS symptomatiques [4] mais aussi les AAS asymptomatiques chez la femme en âge de procréer, les AAS dont la taille est supérieur à 20mm et/ou qui augmentent de taille et enfin les AAS dans un contexte d'hypertension portale [5]. Le traitement des AAS peut être endovasculaire ou chirurgical [9]. Le traitement endovasculaire consiste en une exclusion de l'anévrisme par une endoprothèse couverte ou par une embolisation par coils. Le trajet tortueux de l'artère splénique et/ou la possibilité d'une exclusion incomplète expliquent certains échecs techniques du traitement endovasculaire et la nécessité de procédures secondaires. Chez notre patient, l’échec d'une exclusion de l'anévrisme par embolisation était prévisible puisque l'hématémèse traduisait la présence d'une communication de l'AAS et de la lumière gastrique. Ainsi, dans l'hypothèse d'un risque opératoire élevé, une tentative de traitement endovasculaire par endoprothèse couverte aurait été plus logique puisqu'elle aurait assuré la déconnexion entre les lumières vasculaire et digestive. Le risque d’échec aurait alors été associé au caractère relativement distal de l'anévrisme rendant difficile le largage adéquat de la prothèse et au risque objectif de contamination septique de la prothèse attesté par la présence de micro abcès dans la paroi anévrismale. Quant au traitement chirurgical des AAS, il consiste le plus souvent en une résection ou une mise à plat de l'anévrisme sans rétablissement de continuité artérielle [10] et sans splénectomie. Une splénectomie emportant l'anévrisme paraît plus adaptée en cas d'AAS enchâssé dans le hile splénique. En cas d'AAS rompu dans le tube digestif, la cure de l'anévrisme est complétée par une suture digestive. Une approche laparoscopique des AAS est envisageable à l'exception des formes rompues et/ou de grande taille (>5cm) pour lesquelles le contrôle vasculaire notamment de l'artère afférente peut être délicat [4, 5]. Pour notre patient, le caractère rompu de l'AAS indiquait de façon formelle une cure chirurgicale urgente par voie conventionnelle. Dans cette observation, l'embolisation d'un AAS rompu dans l'estomac a été une cause de retard thérapeutique associé à un recours évitable à une transfusion sanguine et une cause probable de majoration des phénomènes inflammatoires locaux ayant favorisé la survenue d'une fistule pancréatique postopératoire.

## Conclusion

La rupture dans l'estomac d'un anévrisme de l'artère splénique (AAS) est une cause rare d'hémorragie digestive. Le diagnostic positif et la configuration anatomique de l'AAS sont établis efficacement par l'angioscanner abdominal. Après avoir stabilisé l’état hémodynamique et éliminé une cause intraluminale potentiellement contrôlable endoscopiquement, le traitement de référence est la cure chirurgicale de l'AAS par voie conventionnelle sans rétablissement de la continuité artérielle splénique, sans splénectomie et avec suture de l'orifice digestif.
